# Modestly degraded microarchitecture and high serum levels of osteopontin in Swedish females with anorexia nervosa

**DOI:** 10.1007/s40519-020-01062-8

**Published:** 2020-11-06

**Authors:** Pär Wanby, Lars Brudin, Siv-Ping Von, Martin Carlsson

**Affiliations:** 1 Department of Medicine and Optometry, Linnaeus University, SE, 391 82 Kalmar, Sweden; 2 Department of Medical and Health Sciences, University of Linköping, SE, 581 83 Linköping, Sweden; 3 Department of Internal Medicine, Section of Endocrinology, Region Kalmar County, 392 44 Kalmar, Sweden; 4 Department of Medical and Health Sciences, University of Linköping, SE, 581 83 Linköping, Sweden; 5 Department of Clinical Physiology, Region Kalmar County, SE, 392 44 Kalmar, Sweden; 6 Department of Clinical Chemistry, Region Kalmar County, SE, 392 44 Kalmar, Sweden; 7 Department of Medicine and Optometry, Linnaeus University, SE, 391 82 Kalmar, Sweden

**Keywords:** Anorexia nervosa, Microarchitecture, TBS, Osteopontin, Dkk-1

## Abstract

**Purpose:**

Adult women with long-time anorexia nervosa (AN) are believed to have osteopenia (T-score ≤ 1.0) in 93 % and osteoporosis (T-score ≤ 2.5) in 38 %. Bone microarchitecture assessed by Trabecular Bone Score (TBS) predicts osteoporotic fractures. Our aim was to evaluate the microarchitecture in adult females with AN by determining TBS and to identify factors potentially associated with TBS, such as bone turnover markers.

**Methods:**

20 female patients with AN (DSM IV), aged 27.8 ± 4.4 years, BMI 16.6 ± 0.6 kg/m^2^ and duration of illness of 8.5 ± 5 years had previously been evaluated with dual-energy X-ray absorptiometry (DXA). TBS measurements were now obtained, using iNsight software, from spinal DXA images. Serum levels of bone turnover markers were determined in patients and healthy normal-weight controls.

**Results:**

Compared to controls serum values of osteopontin were higher (*p *= 0.009). BMD in patients with AN was reduced by at least 1.0 SD at one or more skeletal sites in 65 % of patients and by at least 2.5 SD in 20 %. Only one of the patients (5%) had suffered a fracture. TBS (mean 1.35 ± 0.06; median 1.36 (1.23–1.44) was in the lower normal range (≥ 1.35). 40 % of patients showed partially (> 1.20 and < 1.35) but none showed a fully degraded micro-architecture.

**Conclusions:**

In Swedish AN patients we found a low reduction of BMD and fracture history. The bone microarchitecture, evaluated for the first time for this group by TBS, was only modestly compromised, and to a lesser extent than expected for this group of patients with AN.

**Level of evidence:**

Level V; cross-sectional descriptive study.

## Introduction

Anorexia nervosa (AN) is characterized by low-fat mass, often complicated by reduction of bone mass, impaired bone structure, and fractures [1]. As an adaptation to chronic undernutrition, multiple endocrine abnormalities develop with a profound impact on bone metabolism, and a compromised bone mineral density (BMD) is a hallmark of AN [2]. Adult women with long-term AN [3] are believed to have osteopenia (T-score ≤ 1.0) in 93 % and osteoporosis (T-score ≤ 2.5) in approximately 40 %. When AN develops during adolescence peak bone accrual is often suboptimal [4] and BMD tends to be even lower than in those who develop AN in adulthood, despite a similar duration of amenorrhea [5]. However, regardless of age, fracture risk is increased for this condition and is also increased in women with a past history of AN [1, 6].

BMD is measured by dual-energy X-ray absorptiometry (DXA) and is a major determinant of fracture risk [4], although BMD only explains 60–80 % of all fractures in postmenopausal females [7, 8]. Skeletal factors other than BMD, such as microarchitecture, also contribute to bone strength and fracture risk. In adolescent girls with AN, as well as in adult women with AN, impaired microarchitecture has been demonstrated with flat-panel ultra-high-resolution volume computed tomography [9]. Trabecular bone score (TBS) is a grey-level textural index that captures elements of three-dimensional bone architecture such as trabecular number and separation that are not obtained in a standard DXA measurement [10, 12], but obtainable from spinal DXA data. TBS is thus an easily accessible, non-invasive method that offers information beyond that which DXA BMD measurements provide, and in post-menopausal women TBS predicts fracture risk [11]. Evidence of degraded bone microarchitecture, expressed as reduced levels of TBS, has been demonstrated in adolescent girls [13, 14], but not, to our knowledge, in adult patients with AN.

The aim of this study was to evaluate the microarchitecture in a group of adult Swedish females with AN, by determining TBS. A secondary aim was to identify clinical and biochemical factors, such as bone turnover markers, and their potential association with TBS.

## Methods

Twenty female patients with AN (DSM IV), aged 27.8 ± 4.4 years, with a BMI of 16.6 ± 0.6 kg/m^2^ and a duration of illness of 8 years (0.5–21), had been previously included in a vitamin D study (2013-16) at an outpatient clinic in southeastern Sweden specializing in the treatment of eating disorders [15], and at inclusion were evaluated with DXA (GE Lunar iDXA). Following the availability of TBS measurements the patients were included in the present study, and TBS measurements were obtained retrospectively from spinal DXA images of the patients with AN, using iNsight software. Neither patients on medication nor with a chronic disease affecting bone health, nor pregnant patients were included in the study [15].

BMD was determined by dual emission X-ray absorptiometry (DXA) of the total hip and lumbar region (GE Lunar iDXA (GE Healthcare Lunar, Madison, WI), with a precision of 0.007 g/cm^2^ (CV = 0.5%) [16]. TBS values ≥ 1.35 were considered normal, > 1.20 and < 1.35 partially degraded, and ≤ 1.20 degraded microarchitecture [7, 13].

Bone turnover markers were assessed in the twenty patients with AN and in a control group consisting of 78 healthy female blood donors (age 22.6 ± 2.0 years, BMI 24.9 ± 4.7 kg/m^2^) that volunteered for the study. This healthy control group has also been previously presented [15].

All participants had given their written informed consent to participate in the study, which had received the approval of the Regional Ethical Committee, Linköping, Sweden.

### Assays

Non-fasting venous blood samples were drawn for analysis. Free vitamin D (25(OH)D) was determined by an ELISA method (Future Diagnostics Solutions B.V, Netherlands). For details regarding assays determining total serum 25(OH)D, plasma calcium, serum ionized calcium, serum PTH, plasma albumin, plasma creatinine, and serum leptin, see reference [15].

Serum testosterone, Luteinizing Hormone (LH), Follicle-Stimulating Hormone (FSH) plasma Thyroid-Stimulating Hormone (TSH) and free plasma tyroxin (T4) were analyzed using the instrument Cobas e601 electrochemiluminescence immunoassays from Roche Diagnostics, Basel, Switzerland. Blood samples were analyzed for plasma alkaline phosphatase (ALP) using the instrument AU680, Beckman Coulter, Brea, CA, USA.

Bone turnover marker analysis (Dickkopf-1 [Dkk-1], Osteoprotegerin [OPG], Osteocalcin, and Osteopontin [OPN] was performed simultaneously according to Merck Millipore’s instructions for the xMAP technology with multiplex beads. Plates (Human Bone Magnetic Bead Panel from Merck Millipore) were measured using the Luminex’s xMAP® instrument MagPix LX 200 (Luminex, Austin, TX, USA). All samples were analysed in duplicates. If duplicate results differed by more than 20 % measurements were repeated on a second aliquot. The controls were analysed in the beginning and end of each plate. The coefficients of variation (% CVs) were for Dkk-1: 2,7; OPG: 2,7; osteocalcin: 6,3; OPN: 8,3.

### Statistical analysis

All continuous variables are presented as the mean (SD) and median (range). Student´s test or the Chi-square test was used to compare serum values of bone turnover markers in patients with anorexia and controls. Correlations between trabecular bone score (TBS) and other various variables assessed at study inclusion were calculated using Spearman´s non-parametric regression. Statistical analyses were performed with STATISTICA (version 12, Statsoft^®^, Tulsa, USA), and p-values < 0.05 were considered statistically significant.

## Results

Clinical characteristics of the 20 patients with AN are summarized in Table 1.

**Table 1:** Clinical characteristics in 20 patients with anorexia nervosa (AN); previously in part published by Carlsson et al [15]

Variable	
*N*	20
Age at baseline (years)	
Mean (SD)	27.8 (4.4)
Median (range)	28 (21–36)
Age at menarche (years)	
Mean (SD)	13.7 (2.1)
Median (range)	13 (11–19)
Duration of anorexia (years)	
Mean (SD)	8.5 (5.0)
Median (range)	8.0 (0.5–21.0 )
BMI (kg/m^2^)	
Mean (SD)	16.6 (0.6)
Median (range)	16.7 (15.5–17.7)
Plasma calcium (mmol/L)	
Mean (SD)	2.4 (0.1)
Median (range)	2.4 (2.2–2.5 )
Serum-ionized calcium (mmol/L)	
Mean (SD)	1.26 (0.05)
Median (range)	1.28 (1.12–1.38)
Serum PTH (pmol/L)	
Mean (SD)	3.7 (1.7)
Median (range)	3.6 (2.0–9.7 )
Total serum 25(OH)D (nmol/L)	
Mean (SD)	79.3 (32.2)
Median (range)	80 (22–165)
Free serum25(OH)D (nmol/L)	
Mean (SD)	6.5 (2.5)
Median (range)	6.6 (1.9–11.6 )
Plasma albumin (mmol/L)	
Mean (SD)	44.3 (4.0)
Median (range)	45 (35–50)
Plasma-creatinine (µmol/L)	
Mean (SD)	64.3 (8.9)
Median (range)	65 (46–79)
Serum testosterone (nmol/L)	
Mean (SD)	0.8 (0.4)
Median (range)	0.7 (0.4–1.7 )
Serum luteinizing hormone (LH IU/L)	
Mean (SD)	3.2 (6.3)
Median (range)	0.5 (0.1–27.0 )
Serum follicle-stimulating hormone (FSH (IU/L)	
Mean (SD)	4.4 (3.7)
Median (range)	4.7 (0.4–14.0 )
Plasma thyroid-stimulating hormone (TSH mIE/L)	
Mean (SD)	1.9 (0.9)
Median (range)	1.7 (0.7–4.2 )
Free plasma tyroxin (T4 pmol/L)	
Mean (SD)	13.5 (1.9)
Median (range)	13.5 (10.0–17.0)
Plasma-ALP (µkat/L)	
Mean (SD)	0.96 (0.24)
Median (range)	0.9 (0.6–1.4)
Bone mineral density hip (g/cm^2^)	
Mean (SD)	0.85 (0.13)
Median (range)	0.86 (0.57–1.07)
T-score Hip	
Mean (SD)	– 1.26 (1.09)
Median (range)	– 1.17 ( – 3.58 to 0.59)
Z-score Hip	
Mean (SD)	– 0.83 (1.08)
Median (range)	– 0.67 ( – 3.15 to 1.15)
Bone mineral density lumbal (g/cm^2^)	
Mean (SD)	0.99 (0.09)
Median (range)	1.01 (0.78–1.12)
T-score_lumbal	
Mean (SD)	– 1.77 (0.78)
Median (range)	– 1.61 ( – 3.54 to – 0.67)
Z-score lumbal	
Mean (SD)	– 1.01 (0.75)
Median (range)	– 0.87 ( – 2.84 to 0.13)
Trabecular bone score (TBS)	
Mean (SD)	1.35 (0.06)
Median (range)	1.36 (1.23 - 1.44)
BMD hip category (*n*, %)	
Osteoporosis	3 (15.0)
Osteopenia	8 (40.0)
Normal	9 (45.0)
BMD lumbal category (*n*, %)	
Osteoporosis	4 (20.0)
Osteopenia	13 (65.0)
Normal	3 (15.0)
Osteoporosis	4 (20.0)
Osteopenia	13 (65.0)
Normal	3 (15.0)

Patients with AN had low values of hip bone mineral density (BMD; 0.85 ± 0.13 g/cm^2^, T-score – 1.26 ± 1.09, Z-score −0.83 ± 1.08) and lumbar spinal BMD (0.99 ± 0.09 g/cm^2^, T-score −1.77 ± 0.78 and Z-score −1.01 ± 0.75). 40 and 65 % in the hip and lumbar spine, respectively, were osteopenic, 15 and 20 % were osteoporotic, respectively (Table 1). BMD was reduced by at least 1.0 SD at one or more skeletal sites in 65 % of patients and by at least 2.5 SD in 20 % of patients. Only one of the patients (5%) had suffered a fracture.

Serum values for leptin and bone turnover markers in patients with AN and controls are presented in Table 2. Serum values for leptin (*p*>0.001) and DKK-1 (*p*=0.047) were lower in patients with AN while serum values for osteopontin were higher (*p*=0.009).

**Table 2:** Serum leptin and bone turnover markers in patients with anorexia nervosa (AN) and controls

Variable	Anorexia	Controls	Diff (*p*)
*N*	20	78	–
Age at baseline (years)			
Mean (SD)	27.8 (4.4)	22.6 (2.0)	
Median (range)	28 (21–36)	22 (19–26)	< 0.001
BMI-baseline (kg/m^2^)			
Mean (SD)	16.6 (0.6)	24.9 (4.7)	
Median (range)	16.7 (15.5 - 17.7 )	24.2 (18.1 - 39.6)	< 0.001
Dickkopf-1 (Dkk-1; ng/L)			
Mean (SD)	2247.4 (684.0)	2596.5 (737.0)	
Median (range)	1937 (1302–3692)	2506 (1203–5603)	0.047
Osteoprotegerin (OPG; ng/L)			
Mean (SD)	522 (106)	481 (142)	
Median (range)	508 (389–786)	455 (250–1130)	0.041
Osteocalcin (µg/L)			
Mean (SD)	25739 (12757)	25972 (9209)	
Median (range)	20162 (8570–58986)	24298 (11890–64063)	0.369
Osteopontin (OPN; µg/L)			
Mean (SD)	13297 (4337)	10461 (5773)	
Median (range)	13364 (5618–24250)	9261 (1990–36547)	0.009
Leptin (ng/ml)			
Mean (SD)	2.9 (2.3)	25.9 (21.7)	
Median (range)	2.7 (0.0–8.2 )	20.5 (0.5–120.1)	< 0.001

TBS (mean 1.35 ± 0.06; median 1.36 (1.23–1.44) was in the lower normal range (≥ 1.35). 40 % of patients showed partially (> 1.20 and < 1.35) degraded microarchitecture. None showed a fully degraded microarchitecture.

TBS was negatively associated with the age of menarche (*p*=0.008) and positively associated with BMI (*p*=0.017), BMD of the hip (*p*=0.013), hip T- and Z-scores (*p*=0.013 and *p*=0.010, respectively) but not with the duration of AN or BMD of the lumbar spine (*p*=0.110), nor with any of the bone turnover markers (Table 3).

**Table 3:** Correlations between trabecular bone score (TBS) and the other various variables assessed at study inclusion using Spearman´s non-parametrsic regression

Variable	Trabecular bone score (TBS)		
	*N*	*R*	*p*
Age at baseline (years)	20	– 0.150	0.528
Age at menarche (years)	18	– 0.602	0.008
Duration of anorexia (years)	19	0.054	0.827
BMI (kg/m^2^)	20	0.527	0.017
Plasma calcium (mmol/L)	19	0.346	0.147
Serum-ionized calcium (mmol/L)	19	0.363	0.126
Serum PTH (pmol/L)	19	– 0.026	0.917
Total serum 25(OH)D (nmol/L)	19	– 0.174	0.477
Free serum 25 (OH)D (pg/ml)	18	– 0.099	0.695
Plasma albumin (mmol/L)	19	0.298	0.215
Plasma-creatinine (µmol/L)	19	– 0.016	0.949
Serum testosterone (nmol/L)	19	– 0.047	0.847
Serum luteinizing hormone (LH IU/L)	19	0.193	0.430
Serum follicle-stimulating hormone (FSH (IU/L)	19	0.091	0.711
Plasma thyroid-stimulating hormone (TSH mIE/L)	19	0.500	0.029
Free plasma tyroxin (T4 pmol/L)	18	– 0.034	0.895
Plasma-ALP (µkat/L)	18	0.035	0.891
Bone mineral density hip (g/cm^2^)	20	0.547	0.013
T-score Hip	20	0.547	0.013
Z-score Hip	20	0.562	0.010
Bone mineral density lumbar spine (g/cm^2^)	20	0.368	0.110
T-score lumbar spine	20	0.368	0.110
Z-score lumbar spine	20	0.268	0.254
Leptin (ng/ml)	17	– 0.015	0.955
Dickkopf-1 (Dkk-1; ng/L)	20	0.001	0.997
Osteoprotegerin (OPG; ng/L)	20	– 0.181	0.444
Osteocalcin (µg/L)	20	– 0.141	0.552
Osteopontin (OPN; µg/L)	20	0.018	0.940

## Discussion

In this study, conducted in a small population of young Swedish female adults with a long duration of AN we assessed microarchitecture using TBS. The major finding was only a modestly compromised TBS in accordance with an unexpectedly low reduction of BMD and fracture history.

The utility of TBS has been evaluated in several populations studies [10–12, 17–20], but to the best of our knowledge this is the first study to test the practicality of deriving TBS from lumbar spinal DXA scans to assess microarchitecture in adult patients with AN. Previously, this has been tested in numerous populations but in AN only among adolescent patients [13, 14]. TBS was, in these two studies, partially degraded in 33 % and 59 %, and degraded in an additional 11 % and 8 %, respectively. The mean patient age of both these studies was about 15.5 years with an illness duration of 4 months and 2 years, a mean BMI of 18.9±1.8 and 15.9±2.2 kg/m^2,^ respectively. In our study of young adult females with a mean BMI of 16.6±0.6 kg/m^2^, 40 % had a partially degraded microarchitecture despite a much longer duration of AN. The more pronounced reduction in TBS in the later study of adolescents with AN may be due to a lower BMI, or to the more deleterious effects of AN on the bone when encountered during bone mass acquisition as to when AN develops in adulthood [4, 5]. It is uncertain whether this also translates to bone microarchitecture.

In both aforementioned studies of paediatric patients with AN [13, 14], numerous correlations with anthropomorphic and clinical variables were demonstrated. In our study, we found an association between TBS with the age of menarche, BMI, and DXA measures of the hip, but not with the lumbar spine. The low number of patients could have contributed to the lack of association to lumbar spine parameters as to a previously demonstrated association between trabecular microarchitecture and disease duration [21]. Leptin and testosterone, for which we found no association to TBS, has furthermore been demonstrated to, independent of BMI, predict bone microarchitecture [22].

In an often-cited study by Grinspoon and colleagues BMD was, in women with a mean age of 24 years, a mean duration of AN of more than 5 years, and a mean BMI of 17.1 kg/m^2^, reduced by at least 1.0 SD at one or more skeletal sites in 92 % of patients, and by at least 2.5 SD in 38 % [3]. In a subsequent study by Miller and co-workers in patients with a similar patient age and illness duration (mean BMI 16.8 kg/m^2^), corresponding percentage figures were 86 and 34, respectively [23]. In our patients BMD was to a lesser degree compromised; 65 % were either osteopenic or osteoporotic and 20 % osteoporotic. Our finding of a less reduced BMD in adult patients (mean age 27.8±4.4) with AN compared to previous studies can most likely be explained by our small study population, and an unexpectedly low fracture incidence, 5 % in comparison to 30 % [24], supports this line of reasoning. On the other hand, although small, our group of patients with a long duration of illness and a rather low BMI represents a sample with, in many respects, relatively severe AN. Parameters of bone health were thus expected to be likewise, which makes our findings unexpected. Moreover, the cited studies [3, 23] were performed at the beginning of this century, and more recent studies of BMD in adult patients with AN are scant. Larger studies in adult patients with AN are thus warranted to clarify representative levels of BMD affection as well as the extent of TBS degradation and the potential role of TBS in fracture prediction for this condition.

In this study, we measured several bone turnover markers previously not fully characterized in AN, nor in relation to bone architecture in particular. We could not demonstrate any correlations between TBS and bone turnover markers, but as anticipated we found decreased serum levels of the Wnt signalling pathway inhibitor, DKK-1 [25]. For OPG we measured increased serum levels, which confirms a finding by Ostrowska et al [26], although in patients with AN an increased secretion of RANKL, and decreased levels would be expected due to estrogen deficiency [27].

Osteopontin (OPN) is a non-collagenous matrix protein expressed in bone tissue, which is involved in bone remodeling and capable of activating bone resorption [28]. Osteopontin has been associated with increased fracture risk in postmenopausal women, but previous reports on serum levels in subjects with AN are lacking. Our finding of highly increased levels of Osteopontin in AN are expected but may represent a novel finding requiring further research.

Some limitations of the study must be acknowledged. Foremost, our group of patients was small and consisted of only female participants, which limits the generalizability of the study, and may explain the lack of associations with various secondary variables such as lumbar spinal DXA values and serum levels of bone turnover markers. Furthermore, we did not obtain DXA scans and, consequently, no TBS values from healthy controls. Instead, we compared TBS measurements with healthy postmenopausal women, which may result in an underestimation of the extent of the microarchitectural degradation in young adult females with AN.

## Conclusions

In adult patients with AN, bone microarchitecture, evaluated in this group for the first time by TBS, was only modestly compromised and did not correlate to a novel finding of increased serum levels of osteopontin. These findings merit larger studies to elucidate the potential role of TBS and bone turnover markers for fracture prediction for this condition.

What is already known on this subject:

Anorexia nervosa (AN) is often complicated by reduction of bone mass, impaired bone structure, and fractures. Microarchitecture, which adds complementary information on fracture risk can be assessed by determining Trabecular Bone Score (TBS). This has previously been done in adolescent girls with AN. Traditional bone turnover markers have also been studied in patients with AN and may add additional information on bone health.

What does this study add:

Evidence of degraded bone microarchitecture, expressed as reduced levels of TBS, has been demonstrated in adolescent girls but has not to our knowledge previously been assessed in adult patients with AN. This study demonstrates the possibility of also using TBS in adult patients with AN and it raises the question if AN of today necessarily is associated with an impaired bone structure to the extent previously believed. The study also demonstrates a possible novel finding of increased serum levels of osteopontin.

## Data Availability

Additional data can on request be made available.
